# What Is the Impact of Multimodal Treatment in Patients with Leiomyosarcoma of Bone? A Multicenter Study of 35 Patients with an Ultra-Rare Tumor Entity

**DOI:** 10.3390/cancers16091633

**Published:** 2024-04-24

**Authors:** Maya Niethard, Carolin Knebel, Andreas Leithner, Per-Ulf Tunn, Janosch Schoon, Peter Reichardt, Athanasios Pogkas, Joanna Szkandera, Daniel Pink, Dimosthenis Andreou

**Affiliations:** 1 Department of Orthopedic Oncology, Sarcoma Center, Helios Klinikum Berlin-Buch, 13125 Berlin, Germany; per-ulf.tunn@helios-gesundheit.de; 2 Center for Orthopedics, Trauma Surgery and Rehabilitation Medicine, University Medicine Greifswald, Ferdinand-Sauerbruch-Straße, 17475 Greifswald, Germany; janosch.schoon@med.uni-greifswald.de; 3 Department of Orthopedics and Sports Orthopedics, Klinikum Rechts der Isar, Technical University of Munich, 81675 Munich, Germany; carolin.knebel@mri.tum.de; 4 Department of Orthopedics and Trauma, Medical University of Graz, 8036 Graz, Austria; andreas.leithner@medunigraz.at (A.L.); dimosthenis.andreou@medunigraz.at (D.A.); 5 Department of Oncology, Sarcoma Center, Helios Klinikum Berlin-Buch, 13125 Berlin, Germany; peter.reichardt@helios-gesundheit.de; 6 Department of Hematology, Oncology and Palliative Medicine, Vivantes Klinikum Berlin Neukölln, Rudower Straße 48, 12351 Berlin, Germany; athanasios.pogkas@vivantes.de; 7 Division of Clinical Oncology, Department of Internal Medicine, Medical University of Graz, 8036 Graz, Austria; joanna.szkandera@medunigraz.at; 8 Department of Oncology, Sarcoma Center Berlin-Brandenburg, Helios Klinikum, 15526 Bad Saarow, Germany; 9 Department of General Orthopedics and Tumor Orthopedics, Münster University Hospital, 48149 Münster, Germany

**Keywords:** leiomyosarcoma of bone, bone sarcoma, systemic chemotherapy, surgical treatment, patient outcome, rare primary malignant bone sarcoma (RPMBS), ultra-rare sarcoma, multimodality treatment, non-osteosarcoma malignant bone tumors

## Abstract

**Simple Summary:**

Primary leiomyosarcoma of bone constitutes less than 0.7% of all primary malignant bone tumors. Currently, there is no consensus on whether therapeutic approaches should align with the biological characteristics seen in soft tissue leiomyosarcoma or be tailored to the bone location. The efficacy of perioperative chemotherapy for this rare tumor type remains uncertain. Our study aimed to assess various treatment modalities across multiple centers. Surgical intervention emerged as the most significant prognostic factor for patient survival in our analysis, with tumors situated axially, indicating poorer prognosis. A considerable portion of patients experienced secondary metastases. Additionally, the perioperative chemotherapy regimens administered did not correlate with enhanced survival outcomes. Hence, the efficacy of perioperative chemotherapy in bone leiomyosarcoma warrants further investigation, alongside the identification of appropriate agents for treatment.

**Abstract:**

Primary leiomyosarcoma of bone (LMSoB) is extremely rare, comprising only <0.7% of primary malignant bone tumors, and is therefore considered an ultra-rare tumor entity. There is currently no consensus as to whether therapeutic strategies should be based on the biological characteristics of soft tissue leiomyosarcoma or on primary tumor localization in the bone. The use of perioperative chemotherapy and its effectiveness in this rare tumor entity remains unclear. We aimed to evaluate the impact of different treatment approaches in a multicenter setting with a total of 35 patients included. The 5-year overall survival (OS) was 74%. Patients with localized disease undergoing surgery had a significantly higher 5-year OS compared to patients who did not undergo surgical treatment (82% vs. 0%, *p* = 0.0015). Axial tumor localization was associated with worse event-free survival (EFS) probability (*p* < 0.001) and OS (*p* = 0.0082). A high proportion of our patients developed secondary metastases. Furthermore, the perioperative chemotherapy protocols applied to our patients were not associated with an improved EFS or OS. Therefore, the benefit of perioperative chemotherapy in LMSoB needs to be further investigated, and the choice of agents still needs to be clarified.

## 1. Introduction

Primary leiomyosarcoma of bone (LMSoB) is an ultra-rare tumor, first described as a separate entity in 1965 [1] and representing <0.7% of primary malignant bone tumors [2]. According to the proposal of the Surveillance of Rare Cancers in Europe (RARECARE) project, tumors with an incidence of less than two per 100,000 are defined as rare diseases [3]. The definition of ultra-rare tumors (incidence of one or fewer per 1,000,000) was consented to by the Connective Tissue Oncology Society in 2020 [4]. It mostly affects the lower limb and predominantly middle-aged patients [5]. Local X-rays show an osteolytic and destructive bone lesion without tumor-associated matrix production [6]. The tumor cells show a smooth muscle differentiation [7], but histopathological diagnosis can be difficult. However, advances in immunohistochemistry have made diagnosis more accurate, especially regarding the discrimination from primary undifferentiated pleomorphic sarcoma (UPS) of bone [8,9].

The histopathological picture of the tumor cells of LMSoB corresponds to that of soft tissue leiomyosarcoma (STLMS), although the tumor cells are primarily found in the bone. It is controversial whether the therapeutic strategy should be based on biological characteristics, i.e., as in STLMS, or on its localization in the bone. This question cannot be answered conclusively at present. The published evidence does not support the same treatment as for a primary malignant bone tumor and reflects the uncertainty of chemotherapy (CTX) regimens [5,10,11,12].

Surgical resection remains the cornerstone of the treatment of LMSoB [11,13]. However, several smaller series have shown that patients with high-grade tumors undergoing surgical treatment only have a high rate of secondary metastases predominantly involving the lung, indicating the urgent need for systemic treatment options [5,13,14,15,16,17]. While the use of perioperative chemotherapy has greatly improved the prognosis of patients with other high-grade bone sarcomas [18,19,20] and has been associated with improved survival of patients with extremity STLMS [21], little is known about its efficacy in LMSoB, and available data are limited and inconsistent. About 20–75% of patients are reported to undergo systemic perioperative treatment in different studies [11,13,15,22,23,24]. One of the largest series from the Japanese Musculoskeletal Oncology Group retrospectively evaluated 37 out of 48 patients with LMSoB who underwent surgical resection without metastasis at the first visit in uni- and multivariate analysis and found that patients undergoing mostly cisplatin-based perioperative chemotherapy had a 5-year disease-free survival probability of 55%, compared to 28% for patients who received no chemotherapy. With the limited number of patients available for their analysis, this difference did not reach statistical significance (*p* = 0.07) [11]. On the other hand, Antonescu et al. found in a series of 19 patients with high-grade tumors no differences in the survival probability of patients undergoing perioperative, mostly doxorubicin-based chemotherapy and patients undergoing surgery only [24]. Different results are also described in relation to the natural course of disease in patients with LMSoB and the prognostic relevance of distant metastases (DM). While Rekhi et al. report that all eight of their patients developed DM within 12 months of diagnosis, resulting in a dismal prognosis [13], Zumarraga et al. describe a comparable outcome with other bone sarcomas with a 5-year overall survival (OS) probability of 59% [22]. Brewer et al., on the other hand, describe a better survival compared to other bone sarcomas for patients without metastases at diagnosis, which was the only relevant prognostic factor in their series, with a 5-year OS probability of 82%.

These conflicting results create confusion in physicians treating patients with LMSoB. We therefore decided to perform a retrospective multicenter study to analyze the outcome of patients with LMSoB after surgical resection, as well as the impact of chemotherapy on patient survival, and identify possible prognostic factors.

## 2. Materials and Methods

### 2.1. Study Design

We retrospectively reviewed the databases of 4 referral sarcoma centers in Germany and Austria and identified 35 patients with a histologically confirmed, primary LMSoB treated between 1993 and 2018. All centers routinely staged patients to rule out bone metastasis from STLMS or from uterine leiomyosarcoma in female patients. Data regarding patient demographics, tumor characteristics, surgical or multimodal treatment, as well as patient follow-up and disease-specific events were retrospectively retrieved from the patients’ medical files and entered into an electronic database. All tumors had been graded according to the FNCLCC grading system; for the purposes of this analysis, grade II and grade III tumors were classified as high-grade. Diagnosis of local recurrence or distant metastasis was considered an event. All patient data were pseudonymized prior to analysis. The study was conducted in accordance with the Declaration of Helsinki and was approved by the local ethics committee of the Berlin Medical Association under number Eth-KB 2020/0015 on 19 August 2020.

### 2.2. Patient Population and Primary Treatment

Patient demographics and tumor characteristics are presented in Table 1.

Mean patient age at diagnosis was 54 years (range, 19–90 years). The absolute tumor size (longest extension of the tumor in 3 planes) was obtained from pathological analysis and available for 31 patients. It amounted to a median of 75 mm (interquartile range (IQR), 62–105 mm). For 16 resection specimens, the tumor size was <8 cm, and for 15 specimens, it was ≥8 cm. Four out of 35 tumors were graded as G1 (11%), 14 tumors were G2 (40%), and 17 tumors were G3 (49%). The median follow-up was 49 months (IQR, 20–118 months) for all patients and 74 months (IQR, 21–125 months) for surviving patients.

Surgical resection of the primary tumor was performed in 31 of the 35 patients (89%). Twenty patients (65%) underwent megaprosthetic replacement, 3 patients (10%) underwent a biological reconstruction (interposition of iliac crest in 1 patient, fibula interposition in 2 patients), and 5 patients (16%) underwent a resection without reconstruction, while an amputation was performed in 3 patients (10%). Negative surgical margins were achieved in 29 patients (94%), while the remaining 2 patients had microscopically positive margins (6%). Twelve patients (39%) developed postoperative complications, including 4 megaprosthetic infections, 4 mechanical implant failures, and 2 periprosthetic fractures. Among the remaining 4 patients who received no surgical treatment, one patient had an unresectable tumor of the sacrum, 2 patients had primary metastases, and the last patient died 3 days after the diagnostic biopsy.

Of the 4 patients with low-grade tumors, 2 died of their disease 9 and 15 years after their diagnosis, respectively. In one patient, the metastases were histologically confirmed several times and he received a total of 9 different lines of systemic therapy before being discharged home with the best supportive care. In the second patient, the pulmonary metastases were confirmed by imaging. His medical history included urothelial carcinoma. He died of metastatic disease and was considered as DOD for analysis.

Systemic chemotherapy was applied to 16 patients (46%) during their primary treatment, with a median number of 6 cycles (IQR, 3–6 cycles). Chemotherapy was administered to 7 patients (44%) both before and after surgery, 4 patients (25%) preoperatively only, 3 patients (19%) postoperatively only, and the 2 patients with primary metastases underwent palliative chemotherapy (13%). Among the 11 patients who underwent chemotherapy before surgery, 1 patient (9%) had <10% vital tumor cells in the histologic evaluation of the surgical specimen after treatment with doxorubicin/dacarbazine, 2 patients (18%) had 10–50% vital tumor cells after treatment within the EURO-B.O.S.S.-protocol (doxorubicin/cisplatin/ifosfamide +/− MTX) [25], and the remaining 4 patients (36%; 3 of whom were treated within the EURO-B.O.S.S.-protocol, and 1 received doxorubicin/ifosfamide) had >50% vital tumor cells according to the response classification by Salzer-Kuntschik [26]. The respective data on histological response were missing for 4 patients (36%). Eight patients (50%) underwent cisplatin-based chemotherapy, analogous to other spindle-cell bone sarcomas, usually in combination with doxorubicin and ifosfamid +/− methotrexate (according to the EURO-B.O.S.S.- or COSS-96 protocol) [27]. Four patients (25%) had soft tissue sarcoma-like, doxorubicin-based chemotherapy. One patient was administered ifosfamide, carboplatin, and etoposide (Appendix A, Table A1). The last patient without primary metastases received ifosfamide monotherapy in combination with radiotherapy postoperatively outside a referral center, after undergoing surgical treatment with microscopically positive margins. One of the two patients with primary metastases received doxorubicin monotherapy, while the second had a total of 9 different lines of chemotherapy over a period of two years until they died of the disease. Characteristics of patients with high-grade tumors without primary metastasis receiving chemotherapy for primary tumors are depicted in Table 2.

Ten patients (29%) underwent perioperative radiotherapy of a primary tumor; 23 patients (66%) did not. No data were available for 2 patients (6%). Radiotherapy was administered after R0-resection in 7 patients (20%) and in 1 patient (3%) after R1-resection. One patient with a pelvic primary tumor received preoperative radiotherapy and 1 patient underwent radiotherapy in a palliative setting.

### 2.3. Statistical Analysis

Contingency tables were analyzed using the chi-squared test. Continuous variables were checked for normality using the Shapiro–Wilk test. Medians with interquartile ranges (IQR) were calculated for non-normally distributed data and means with ranges for normally distributed data. Non-parametric analyses were carried out with the Mann–Whitney U test. The durations of follow-up and time-to-event (disease progression, locoregional recurrence, distant metastasis, or death) were calculated from the date of the diagnostic biopsy. Survival curves were calculated with the Kaplan–Meier method and compared with the log-rank test. Statistical calculations were performed with the IBM SPSS statistics software version 25.0 (IBM Corp., Armonk, NY, USA) and GraphPad Prism version 8.4.3 (GraphPad Software, Boston, MA, USA). All *p* values were two-sided; a *p* < 0.05 was considered significant.

## 3. Results

After a median follow-up of 49 months (IQR, 20–118 months), the event-free survival probability (EFS) was 66% after 2 years and 50% after 5 years. OS probability was 81% and 74% after 2 and 5 years, respectively. Twenty-one patients (60%) developed distant metastases after a median of 20 months (IQR, 12–63 months). At the last follow-up, 11 patients (31%) were alive with their disease (AWD), 14 patients (40%) were alive with no evidence of disease (NED), and 10 patients (29%) had died (DOD). Thirty-one patients (89%) underwent surgical treatment. Of these, 29 patients (94%) had wide surgical margins. Nine patients (29%) developed surgical complications. Patients with localized disease undergoing surgery had a significantly higher 5-year OS compared to patients who did not undergo surgical treatment (82% vs. 0%, *p* = 0.0015).

**Figure 1:**
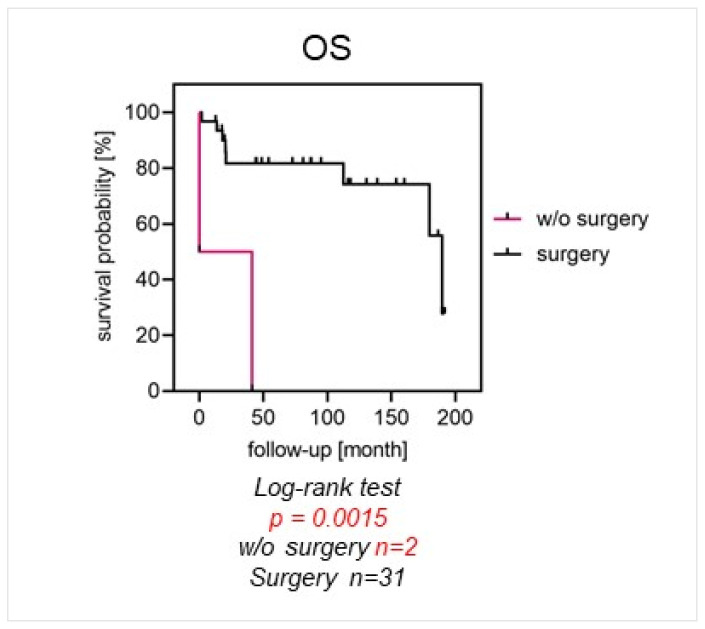
Surgical treatment as prognostic factor: Patients with localized disease undergoing surgery had a significantly better OS of 82% after 5 years, compared to 0% for patients who had no surgery (*p* = 0.0015).

**Figure 2:**
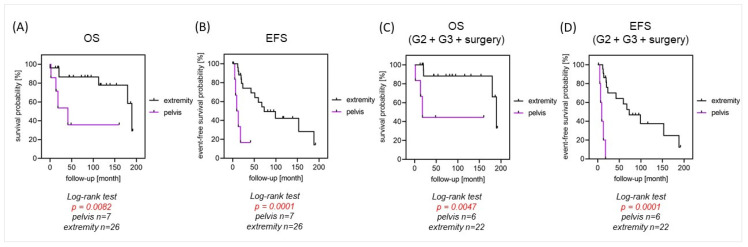
(**A**–**D**) OS and EFS in relation to tumor localization in patients with localized disease.

Patients with localized disease undergoing surgery had a significantly better OS of 82% after 5 years, compared to 0% for patients who had no surgery (*p* = 0.0015) (Figure 1). OS was significantly better for patients with localized disease and tumor localization in the extremities than for pelvic tumor locations (87% vs. 54% and 87% vs. 36% after 2 and 5 years; *p* = 0.0082). Furthermore, they showed a significantly higher 5-year EFS of 59%, compared to 0% (*p* < 0.001), respectively (Figure 2A,B).

High-grade tumors were seen in 31 patients (89%). Two of them presented with primary metastasis (6%). Patients with high-grade tumors of the pelvis undergoing surgical resection had a significantly lower EFS (0% vs. 55%, *p* < 0.001) and OS (60% vs. 100%, *p* = 0.0047), compared to patients with high-grade tumors in the extremities (Figure 2C,D)

Perioperative chemotherapy was given to 15 patients with high-grade tumors without primary metastases (52%). There were no differences in EFS (40% vs. 44% at 5 years, *p* = 0.8459) and OS (80% vs. 73% at 5 years, *p* = 0.4292) between patients with or without chemotherapy.

Four patients (11%) developed a local recurrence after a median time of 28 months (IQR, 12–120 months). The local recurrence probability amounted to 7% after 2 years and 11% after 5 years. Twenty-one patients (60%) developed secondary metastases after a median time of 20 months (IQR, 12–63 months). Of those, eighteen patients (86%) had pulmonary metastases, five patients (24%) had osseous metastases, two patients (10%) had hepatic metastases, one patient (5%) had renal metastasis, and six patients (29%) presented with other metastases, such as skin, soft tissue, or cardiac metastases. Eight of these patients (38%) had metastatic disease in multiple sites. The probability for secondary metastases was 39% after 2 years and 54% after 5 years. EFS probability amounted to 66% after 2 years and 50% after 5-year OS probability amounted to 81% after 2 years and 74% after 5 years.

With respect to survival, there was no statistically significant difference between patients with localized disease and tumor size </> 8 cm with an OS of 85% vs. 73% after 2 years and 77% vs. 73% after 5 years (*p* = 0.2998).

**Figure 3:**
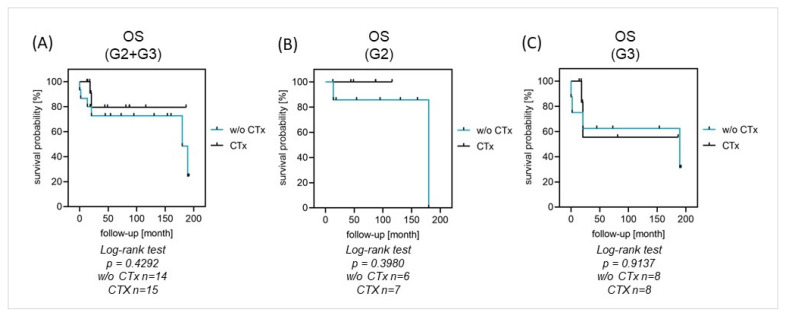
(**A**–**C**) OS in localized disease with and without chemotherapy. Systemic chemotherapy was not associated with differences in survival in high-grade tumors, regardless of the grading.

**Figure 4:**
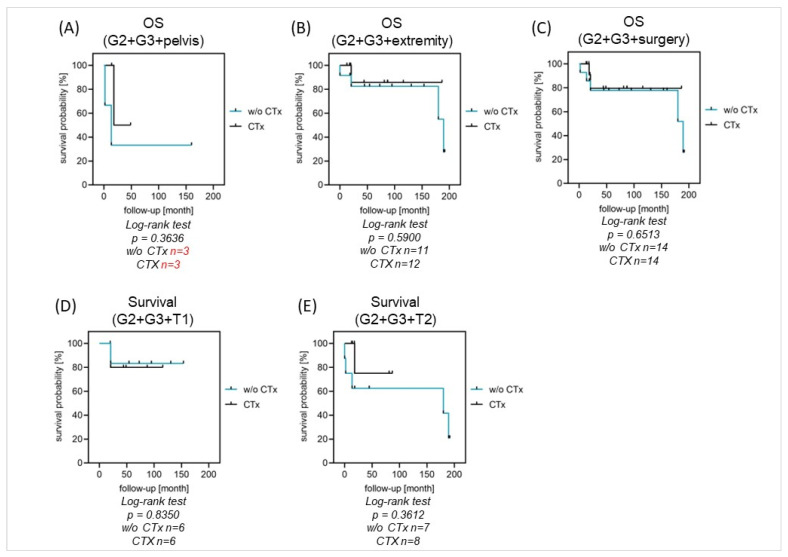
(**A**–**E**) OS in localized disease with and without chemotherapy. Systemic chemotherapy was not associated with differences in survival in high-grade tumors regardless of the tumor location (**A**,**B**), tumor size (**D**,**E**), or after surgical treatment (**C**).

Systemic chemotherapy was not associated with significant differences in OS or EFS in the performed analysis for patients with high-grade tumors and localized disease (n = 29). There was no difference between G2 or G3-graded tumor entities (Figure 3A–C). For patients with tumor resection, there was no difference in survival between the groups with or without chemotherapy (5-year OS: 80% vs. 87%, *p* = 0.6513). From the group of fourteen patients with chemotherapy, two patients died, whereas from the group of fourteen patients without chemotherapy, five patients died (Figure 4A–E). Given the small number of patients and the number of different chemotherapy protocols that were applied, we were not able to perform comparative analyses on the efficacy of the various protocols.

Regarding other possible prognostic factors, patients with high-grade tumors had an OS of 74% after 5 years, compared to 75% for patients with low-grade tumors (*p* = 0.6216), although it should be noted that two of the four patients with low-grade tumors died of their disease 9 and 15 years after their diagnosis, respectively.

## 4. Discussion

LMSoB represents an ultra-rare and diagnostically challenging primary malignant bone tumor, with a high potential for secondary metastasis predominantly involving the lungs [5,8,9,11]. Although there are many case reports describing LMSoB even in rare skeletal localizations [10,28,29], only a few large case series have been published, and prognostic factors and treatment options are not well-defined.

The 5-year overall survival (OS) probability was significantly higher for patients with localized disease who underwent surgery than for patients who did not receive surgical treatment. Axial tumor localization was found to be associated with a worse probability of EFS and OS. Perioperative chemotherapy protocols applied to the patients did not result in an improved EFS or OS.

Our study confirms the fact that surgery is the mainstay of treatment of LMSoB, associated with a high probability of patient survival. This finding is in line with the results of Gusho et al., who retrospectively analyzed 74 patients with LMSoB documented in the Surveillance, Epidemiology, and End Results (SEER) database from 1975 to 2016. They showed that all documented types of surgical treatment (local excision, partial resection, wide resection with limb salvage, and amputation) were positive prognostic factors in LMSoB [10]. As only two patients in our cohort had positive surgical margins, we were unable to meaningfully evaluate the prognostic significance of this parameter. However, Mori et al. previously demonstrated in a study from the Japanese Musculoskeletal Oncology Group that negative surgical margins were associated with a significantly higher disease-free survival probability, as well as significantly higher OS probability in patients with localized disease at diagnosis [5,11].

Like in previous studies, the patients in our analysis had a high overall risk for distant metastases, with 21 out of 33 patients with localized disease developing systemic recurrences after a median time of 20 months. Previous studies also demonstrated that more than 50% of LMSoB patients go on to develop secondary metastases [14,17].

Back in 1987, Berlin et al. reviewed a series of 16 patients. None of them received chemotherapy for a primary tumor. Of the fourteen patients followed for more than 5 years or until death, eight died of distant metastases, reflecting a 5-year OS of 43% [17]. In a case series by Rekhi et al. evaluating eight patients with LMSoB, all eight patients developed distant metastases regardless of the tumor grade within the first 12 months after diagnosis [13].

With only two of our 35 cases presenting with primary metastases (6%), we had a slightly lower percentage of primary metastasis compared to other studies [5].

A recent study by Gusho et al. from 2021 analyzing 74 LMSoB cases describes distant metastasis at presentation as a negative prognostic factor [10], thus supporting findings by Brewer et al. from 2012, evaluating a group of 31 patients treated with chemotherapy and surgical resection. Their 5-year disease-free survival (DFS) for all patients was 57%, but for those without metastases at diagnosis (9.6% of all patients) it was 82%, concluding that despite the high incidence of metastases in their cohort, survival for LMSoB without metastases at diagnosis is better than for other bone sarcomas [5].

At the endpoint of our study with a median FU of 74 months, we saw an unexpectedly high rate of 31% of our patients alive with disease. These results are similar to the study by Mori et al. who found a gap between OS and DFS, monitoring that survival of 14 patients with local recurrence or distant metastases was fairly good [11]. We conclude that although LMSoB is described as a highly aggressive tumor, it seems to have a better prognosis for patients without metastasis at presentation and a surprisingly high rate of patients living with their disease after the occurrence of local recurrence and/or distant metastases.

Perioperative chemotherapy was not associated with differences in EFS-probability or OS in our retrospective analysis. However, we must add restrictively that due to the multicenter data collection, it is not comprehensible in retrospect according to which criteria chemotherapy was indicated and why some patients received chemotherapy and others did not. It cannot be ruled out that, for example, highly aggressive tumors were brought to the same level with chemotherapy as less aggressive tumors for which no chemotherapy was given.

There is an inconsistency regarding the time of application (neoadjuvant or adjuvant) and the choice of substances throughout available studies in the literature. Some authors use cisplatin-based chemotherapy, as intended for spindle-cell bone sarcomas (e.g., EURO-B.O.S.S. protocol), while others apply a combination of doxorubicin and dacarbazine as used for STLMS. While Qian et al. could show that surgery plus chemotherapy improves the survival of patients with extremity STLMS [21], supporting data for LMSoB is missing.

In a study by Brewer et al., 58% of patients received chemotherapy. Only 18% (three out of seventeen patients) had >90% necrosis in the resected specimen. Patients with a LMSoB and a pathological response rate to chemotherapy with >90% necrosis tended to have a better survival rate, without the results being statistically significant. Detailed information on the chemotherapy regimen is limited to the observation that patients with high-grade tumors and less than 60 years of age were treated in the same way as osteosarcoma patients during this period. Their conclusion that LMSoB should be treated similarly to osteosarcoma can therefore only refer to recommending chemotherapy in order to get a high percentage of tumor necrosis, but not to the choice of drugs [5]. Of our seven patients with an available histopathologic response classification after neoadjuvant chemotherapy, only one had <10% vital tumor cells in the resection specimen. Remarkably, this patient had received therapy with doxorubicin/dacarbazine corresponding to a therapy concept for STLS. This fact may indicate that, in addition to the localization, the biological aspects of the tumor should also play a role in the selection of the chemotherapy regime.

As the only prospective, uncontrolled study, Palmerini et al. evaluated the use of an osteosarcoma-like chemotherapy regimen, including high-dose methotrexate in the setting of poor histologic response in patients with rare primary malignant bone sarcomas enrolled in the European Over 40 Bone Sarcoma Study (EURO-B.O.S.S; ClinicalTrials.gov number NCT02986503) [12]. The 5-year OS rate for their 20 patients with localized disease of leiomyosarcoma of bone was 54.9% (IQR, 29.5–74.5%), which is slightly lower than the reported 5-year OS rate of 66% for localized high-grade skeletal osteosarcoma from the EURO-B.O.S.S.-group [25], and even lower than the reported 5-year OS rate of 80% in our 15 patients receiving different chemotherapeutical regimens. Due to the standardized therapy protocol, their findings might represent a benchmark to compare to future histology-driven therapeutical approaches.

Another large series was reported by Mori et al. in 2016 from the Japanese Musculoskeletal Oncology Group, where 32 centers contributed their cases, with a total of 48 patients. Sixteen patients received cisplatin-based chemotherapy. Their results showed that just like in our cohort, neoadjuvant chemotherapy was associated with a poor histological response rate, with only one patient being classified as a good responder. They described a 5-year metastasis-free survival (MFS) with cisplatin-based neoadjuvant chemotherapy of 39.8%, while the 5-year MFS without neoadjuvant chemotherapy was 49.6%. The group concluded that chemotherapy could not be proven to have a positive effect on OS and that cisplatin-based chemotherapy may not be appropriate for LMSoB. However, a bias in patient selection cannot be ruled out [11].

In a SEER database analysis (2021), Gusho et al. compared STLMS to LMSoB. Regarding the choice of treatment regime, the study was able to show that both LMSoB and SLMS were more frequently treated surgically. STLMS were more often irradiated, while LMSoB cases more often received chemotherapy (56.8% vs. 19.9%, *p* < 0.001). They described these results as consistent with SLMS being treated as soft tissue sarcoma and PLB possibly as bone sarcoma, but pointed out that the decision could be based on clinical intuition [10].

In summary, in line with our results, none of the mentioned studies could prove a survival benefit for patients with LMSoB treated with chemotherapy. Of the small number of patients for whom information on tumor response is available, a high percentage are classified as non-responders.

The question of which disease group LMSoB should be systematically classified cannot be answered conclusively today. Some centers are more oriented towards the localization of the tumor and primarily assign LMSoB to the group of primary bone sarcomas, while other centers assign LMSoB to the biological group of leiomyosarcomas with one of several possible primary localizations in the bone. From this assignment—in addition to the surgical approach—strategies similar to those used in the treatment of STLMS can be derived (e.g., choice of chemotherapy, use of adjuvant radiotherapy).

In most studies on LMSoB, radiotherapy is not investigated in a structured way. The indication is left to individual therapy decisions like incomplete resections, inoperable tumors, or palliative situations with local recurrence or distant metastases.

Antonescou et al. investigated postoperative adjuvant therapy in LMSoB. This was carried out in 27% of cases and consisted of radiotherapy in 15% of cases and chemotherapy in 12%. No statistical difference in survival was found between patients who received surgery only and patients who received additional chemotherapy and/or radiation [24]. Like in our cohort, none of the published studies have a uniform approach for the use of radiotherapy, and therefore, there are very limited case numbers for comparison. Therefore, recommendations regarding adjuvant therapy for primary LMSoB are difficult to substantiate.

We compared pelvic to extremity tumor localization, detecting a significant benefit regarding OS for patients with high-grade tumors undergoing surgery if the tumor was localized in the extremities. This has also been previously reported by Adelani et al., who found axial skeleton tumors having a decreased OS compared to extremity localization, although they did note that their analysis was confounded by the fact that patients with axial tumors presented more commonly in advanced tumor stages [29].

The great majority of our patients had high-grade tumors, but we could find no differences in OS between high-grade and low-grade tumors, noting that two of the five patients with low-grade tumors died of their disease 9 and 15 years after their diagnosis. Adelani et al. made a similar observation, with four out of fourteen patients (29%) with low-grade tumors dying from their disease after a median survival of 65 months [29]. In contrast, a study by Brewer et al. included five patients with low-grade tumors, none of whom died of their disease in follow-up [5,10]. Similar to our results, Antonescou et al. found no significant differences in DFS or OS probability for low-grade and high-grade tumors, although they did note that this was probably due to the small sample size of the two groups [24]. We therefore conclude that patients with low-grade tumors are not free from the risk of dying from their disease after an acceptable time span of living with their disease.

In many early case reports, LMSoB was often considered to have a poor prognosis; however, with a 5-year OS of 74% in our cohort, the survival rate appears to be comparable to that of other spindle-cell sarcomas. However, the question of whether perioperative chemotherapy has a positive impact on survival and which combination of drugs should be used cannot be answered conclusively.

Therefore, the benefit of chemotherapy in LMSoB needs to be further investigated and the choice of substances still needs to be clarified.

We acknowledge the fact that our study has several limitations. One main limitation is inherent to the retrospective design of our study, leading to a reliance on patient files and the possibility of selection bias. The collection of data from four different centers over a period of 35 years leads to some inhomogeneity in our cohort as well as in the preferred therapeutic approach of each center. However, given the low incidence of LMSoB, multicenter studies of high-volume referral centers are the only way to reach an adequate sample size of patients treated with current gold standards. Our small sample numbers do not allow for a conclusive analysis of all variables, especially in terms of the selected chemotherapeutic protocols, but this is owed to the extraordinary rarity of this primary malignant bone tumor and the lack of reliable data. But compared to other study cohorts on LMSoB, there are only a few studies with larger case numbers.

## 5. Conclusions

Surgical-wide resection remains the mainstay of treatment for this rare entity of primary LMSoB. We demonstrated that patients with tumors located in the extremities have a better prognosis. We could not prove that perioperative chemotherapy is associated with a higher EFS or OS, although definitive conclusions are impossible in a retrospective setting. Since the potential for distant metastasis is high, there is an evident need for systemic treatment options. Despite a high risk for distant metastases, patients seem to have a relatively stable course of survival with their disease. Due to the rarity of LMSoB, further large multi-institutional case series will be needed to answer the question of an optimal multidisciplinary approach.

## Figures and Tables

**Table 1:** Patient demographics and tumor characteristics.

Patients (n = 35)	Value [Range]	Percent [100%]
Sex ratio (M/F)	14/21	40%/60%
Median age (y)	54 [19–90]	
Tumor characteristics (n = 35)		100%
Extremity localization	27	77%
Pelvic localization	8	23%
Size < 8 cm *	16	52%
Size ≥ 8 cm *	15	48%
Grade 1	4	11%
Grade 2	14	40%
Grade 3	17	49%
Pathologic fracture	4	12%
Distant metastasis (n = 23)		100%
Primary metastasis	2	9%
Secondary metastasis	21	91%
Surgery (n = 31)		100%
R0 margin	29	94%
R1 margin	2	6%
Chemotherapy (n = 16)		100%
Preoperative	11	69%
Postoperative	3	19%
Palliative	2	12%
Doxorubicin/Cisplatin/Ifosfamide +/− MTX	8	57%
Doxorubicin + dacarbazin	3	21%
Doxorubicin + ifosfamide	1	7%
Ifosfamide without doxorubicin	2	14%
Tumor response (Salzer-Kuntschik) (n = 7) **		100%
<10% vital cells	1	14%
10–50% vital cells	2	29%
>50% vital cells	4	57%
Radiotherapy (n = 10) ***		100%
Preoperative	1	10%
Postoperative	8	80%
Palliative	1	10%
Median follow up (n = 35)		
All patients (months)	49 [IQR 20–118]	
Surviving patients (months)	74 [IQR 21–125]	

* available in 31 patients. ** available for 7 out of 11 patients who received preoperative chemotherapy. *** available for 33 patients.

**Table 2:** Detailed characteristics for patients with localized disease, with and without CTX.

Patients (n = 33)	+ CTX (n = 14)	− CTX (n = 19)
Size: <8 cm/≥8 cm	5/8	10/6
Grade 1/2/3	0/6/8	4/7/8
Surgery: yes/no	14/0	17/2
Secondary DM: yes/no	8/6	11/8
NED/AWD/DOD	7/5/2	7/4/8
Survival (from date of biopsy)		
− Median EFS in month with (95% CI) − Median OS in month with (95% CI)	20.2 (12.8–81.0)	41.3 (11.8–118.0)
	32.4 (17.7–87.5)	95.1 (20.7–153.7)

CTX = chemotherapy, DM = distant metastasis, NED = no evidence of disease, AWD = alive with disease, DOD = died of disease, EFS = event-free survival in month, OS = overall survival.

## Data Availability

The data presented in this study are available upon request from the corresponding author. The data are not publicly available due to privacy regulations.

## Appendix A

**Table A1:** Detailed characteristics for patients with high-grade tumors without primary metastasis receiving CTX for primary tumor (n = 14).

Patient No.	Tumor Size [mm]	Grading	CTX Combination	CTX Neoadj./Adj.	Regression Grade	Surgical Margin	Status	LR	DM	EFS	FU
1	68	G2	COSS-96	adj.	na	R0	NED	No	No		116
2	62	G3	Euro-B.O.S.S.	adj.	na	R0	DOD	No	Yes	21	21
3	70	G2	Euro-B.O.S.S.	Neoadj	4	R0	NED	No	No		88
4	110	G3	Euro-B.O.S.S.	Neoadj.	4	R0	NED	No	No		81
5	75	G2	Radiation + ifosfamid	adj.	na	R1	AWD	No	Yes	24	44
6	80	G3	Doxorubicin/Dacarbazin	adj.	na	R1	NED	No	No		18
7	135	G3	Doxorubicin/Dacarbazin	Neoadj.	3	R0	NED	No	No		18
8	160	G2	Doxorubicin/Dacarbazin	Neoadj.	nn	R0	AWD	No	Yes	59	87
9	108	G3	Doxorubicin/Ifosfamide	Neoadj.	5	R0	AWD	No	Yes	7	14
10	62	G3	Euro-B.O.S.S.	Neoadj.	6	R0	NED	No	No		20
11	Nn	G3	COSS-96	adj.	na	R0	AWD	No	Yes	73	187
12	80	G2	Euro-B.O.S.S.	Neoadj.	5	R0	AWD	No	Yes	9	49
13	140	G2	Euro-B.O.S.S.	Neoadj.	5	R0	AWD	No	Yes	13	13
14	130	G3	Ifosfamide/Carboplatin/Etoposid	adj.	nn	R0	DOD	yes	Yes	18	18

na = not applicable, nn = not known, CTX = chemotherapy, NED = no evidence of disease, AWD = alive with disease, DOD = died of disease, LR = local recurrence, EFS = event-free survival in month, FU = follow-up in month.

## References

[1] Evans D.M., Sanerkin N.G. (1965). Primary leiomyosarcoma of bone. J. Pathol. Bacteriol..

[2] Siegel R.L., Miller K.D., Jemal A. (2016). Cancer statistics, 2016. CA Cancer J. Clin..

[3] Casali P.G., Trama A. (2020). Rationale of the rare cancer list: A consensus paper from the joint action on rare cancers (jarc) of the european union (eu). ESMO Open.

[4] Stacchiotti S., Frezza A.M., Blay J.Y., Baldini E.H., Bonvalot S., Bovee J., Callegaro D., Casali P.G., Chiang R.C., Demetri G.D. (2021). Ultra-rare sarcomas: A consensus paper from the connective tissue oncology society community of experts on the incidence threshold and the list of entities. Cancer.

[5] Brewer P., Sumathi V., Grimer R.J., Carter S.R., Tillman R.M., Abudu A., Jeys L. (2012). Primary leiomyosarcoma of bone: Analysis of prognosis. Sarcoma.

[6] Ngo A.V., Bartolotta R., Chew F., Linnau K. (2012). Primary skeletal leiomyosarcoma. Radiol. Case Rep..

[7] Jo V.Y., Fletcher C.D. (2014). Who classification of soft tissue tumours: An update based on the 2013 (4th) edition. Pathology.

[8] Wang G.Y., Lucas D.R. (2019). Primary leiomyosarcoma of bone: Review and update. Arch. Pathol. Lab. Med..

[9] Sbaraglia M., Righi A., Gambarotti M., Vanel D., Picci P., Dei Tos A.P. (2017). Soft tissue tumors rarely presenting primary in bone; diagnostic pitfalls. Surg. Pathol. Clin..

[10] Gusho C.A., Blank A.T., Gitelis S. (2021). Comparison of clinicopathological features and outcomes in patients with primary leiomyosarcoma of bone and soft tissue. J. Surg. Oncol..

[11] Mori T., Nakayama R., Endo M., Hiraga H., Tomita M., Fukase N., Kobayashi E., Kawai A., Ueda T., Morioka H. (2016). Forty-eight cases of leiomyosarcoma of bone in japan: A multicenter study from the japanese musculoskeletal oncology group. J. Surg. Oncol..

[12] Palmerini E., Reichardt P., Hall K.S., Bertulli R., Bielack S.S., Comandone A., Egerer G., Hansmeier A., Kevric M., Carretta E. (2023). Outcome of rare primary malignant bone sarcoma (rpmbs) treated with multimodal therapy: Results from the european bone over 40 sarcoma study (euro-b.O.S.S). Cancer.

[13] Rekhi B., Kaur A., Puri A., Desai S., Jambhekar N.A. (2011). Primary leiomyosarcoma of bone—A clinicopathologic study of 8 uncommon cases with immunohistochemical analysis and clinical outcomes. Ann. Diagn. Pathol..

[14] Myers J.L., Arocho J., Bernreuter W., Dunham W., Mazur M.T. (1991). Leiomyosarcoma of bone. A clinicopathologic, immunohistochemical, and ultrastructural study of five cases. Cancer.

[15] Khoddami M., Bedard Y.C., Bell R.S., Kandel R.A. (1996). Primary leiomyosarcoma of bone: Report of seven cases and review of the literature. Arch. Pathol. Lab. Med..

[16] Kameda N., Kagesawa M., Hiruta N., Akima M., Ohki M., Matsumoto T. (1987). Primary leiomyosarcoma of bone. A case report and review of the literature. Acta Pathol. Jpn..

[17] Berlin O., Angervall L., Kindblom L.G., Berlin I.C., Stener B. (1987). Primary leiomyosarcoma of bone. A clinical, radiographic, pathologic-anatomic, and prognostic study of 16 cases. Skelet. Radiol..

[18] Smeland S., Bielack S.S., Whelan J., Bernstein M., Hogendoorn P., Krailo M.D., Gorlick R., Janeway K.A., Ingleby F.C., Anninga J. (2019). Survival and prognosis with osteosarcoma: Outcomes in more than 2000 patients in the euramos-1 (european and american osteosarcoma study) cohort. Eur. J. Cancer.

[19] Gaspar N., Hawkins D.S., Dirksen U., Lewis I.J., Ferrari S., Le Deley M.C., Kovar H., Grimer R., Whelan J., Claude L. (2015). Ewing sarcoma: Current management and future approaches through collaboration. J. Clin. Oncol..

[20] Esiashvili N., Goodman M., Marcus R.B. (2008). Changes in incidence and survival of ewing sarcoma patients over the past 3 decades: Surveillance epidemiology and end results data. J. Pediatr. Hematol. Oncol..

[21] Qian S.J., Wu J.Q., Wang Z., Zhang B. (2019). Surgery plus chemotherapy improves survival of patients with extremity soft tissue leiomyosarcoma and metastasis at presentation. J. Cancer.

[22] Zumarraga J.P., Arouca M.M., Baptista A.M., Caiero M.T., Rubio D.E., de Camargo O.P. (2019). Primary leiomyosarcoma of bone: Clinicopathologic and prognostic factors analysis in a single institution. Acta Ortop. Bras..

[23] Recine F., Bongiovanni A., Casadei R., Pieri F., Riva N., De Vita A., Mercatali L., Liverani C., Spadazzi C., Miserocchi G. (2017). Primary leiomyosarcoma of the bone: A case report and a review of the literature. Medicine.

[24] Antonescu C.R., Erlandson R.A., Huvos A.G. (1997). Primary leiomyosarcoma of bone: A clinicopathologic, immunohistochemical, and ultrastructural study of 33 patients and a literature review. Am. J. Surg. Pathol..

[25] Ferrari S., Bielack S.S., Smeland S., Longhi A., Egerer G., Sundby Hall K., Donati D., Kevric M., Brosjo O., Comandone A. (2018). Euro-b.O.S.S.: A european study on chemotherapy in bone-sarcoma patients aged over 40: Outcome in primary high-grade osteosarcoma. Tumori.

[26] Salzer-Kuntschik M., Delling G., Beron G., Sigmund R. (1983). Morphological grades of regression in osteosarcoma after polychemotherapy—Study coss 80. J. Cancer Res. Clin. Oncol..

[27] Bielack S.S., Kager L., Kuhne T., Langer T., Reichardt P., Blattmann C., Kevric M., Mettmann V., Sorg B., Hecker-Nolting S. (2023). Establishment, maintenance, and performance of the cooperative osteosarcoma study group (coss). Cancers.

[28] Chow L.T. (2016). Metatarsal leiomyosarcoma masquerading as acute osteomyelitis—A diagnostic trap unveiled by vigilant clinical, radiologic and pathologic analysis. Foot.

[29] Adelani M.A., Schultenover S.J., Holt G.E., Cates J.M. (2009). Primary leiomyosarcoma of extragnathic bone: Clinicopathologic features and reevaluation of prognosis. Arch. Pathol. Lab. Med..

